# Supplementary material for: Are carbon emissions trading and green financial instruments synergistic? -Comprehensive quantitative research based on content analysis
Source: PLoS One. 2024 Mar 7;19(3):e0298601. doi: 10.1371/journal.pone.0298601 (PMC10919601; doi:10.1371/journal.pone.0298601)
Supplement: S1 Data — (ZIP) [file pone.0298601.s001.zip › Data/地级市Z_F工作报告.zip!/║╙─╧/╚2├┼╧┐╩╨/└┤╫╘╣1⁄2╓┌║┼í╛┬φ┐╦╩2╛▌═°í┐_2014.docx]

各位代表：

　　现在，我代表市人民政府，向大会报告工作，请予审议，并请各位政协委员和其他列席人士提出意见。

　　一、2013年工作回顾

　　过去的一年，面对错综复杂的经济形势，在省委、省政府和市委的正确领导下，全市上下认真贯彻落实党的十八大和十八届三中全会精神，紧紧围绕“三大战略定位”，深入推进“四大一高”战略，加快转型发展步伐，经济增长速度稳中趋升、质量稳中有进、态势稳中向好。初步测算，全市生产总值达到1205亿元，增长9.1%。全社会固定资产投资1151亿元，增长22.8%，其中工业固定资产投资647亿元，增长23.6%。公共财政预算收入81.8亿元，增长19.2%。新增城镇就业4.2万人；社会消费品零售总额311亿元，增长14%；居民消费价格上涨2.5%。城镇居民人均可支配收入20938元，农民人均纯收入8926元，分别实际增长6.4%和10.5%。单位生产总值能耗下降3.5%，超额完成省定目标。

　　抓项目，促转型，“四大一高”持续深化。把项目建设作为推进“四大一高”战略的重要抓手，实施重点项目307个，完成投资822.5亿元，创历年新高。中金黄金产业园等一批重大项目开工，义煤综能公司1000万标方煤制气及综合利用一期等132个项目建成。大通关方面，三门峡海关投入运行，国家铝质检中心一次性通过“三合一”认证和国家质检总局验收，国家果品与果蔬汁检测重点实验室落户。大交通方面，完成投资70亿元，占年计划的125%。三淅高速灵卢段、郑卢高速洛卢段建成通车。蒙西铁路开工在即，渑垣高速、运宝高速等项目前期加快推进。大商贸方面，大中海商业文化广场、义乌国际商贸城建成开业，华创国际城市综合体等项目稳步推进。第二届特博会成效提升，签订贸易购销合同79亿元，拉动相关产业实现收入21亿元，成为三门峡会展经济的“金字招牌”。大旅游方面，旅游“双十”工程完成投资21.8亿元，增长34.4%，双龙湾景区、甘山国家森林公园通过4A级景区初验。第八届中博会（三门峡分会场）和第19届旅游节暨投洽会盛况空前，旅游区域合作和市场开拓实现新突破。接待国内外游客2054万人次，增长16.9%，旅游总收入176.5亿元，增长17.6%。高新产业方面，实施重大项目66个，完成投资84.6亿元，新认定国家级高新技术企业8家，新增院士工作站3家、市级工程技术研究中心7家。高新产业完成增加值55.4亿元，增长23.7%。荣获“全国科技进步先进市”称号。

　　稳增长，调结构，工业经济平稳向好。深入实施工业稳增长调结构百日攻坚行动，积极开展产销对接、银企对接，及时解决经济运行中的突出矛盾和问题，工业增速逐季加快，规模以上工业实现增加值684亿元，增长10.4%。围绕五大千亿元产业集群，实施亿元以上工业项目82个，完成投资160亿元，鸿宇电子环保型覆铜板等40个项目建成投产。产业集聚区建设稳步推进，完成工业投资428.8亿元，增长35.4%。三门峡产业集聚区跨入省级高新区行列，被确定为国家新型工业化产业示范基地，义马市煤化工产业集聚区进入全省“十强”。

　　打基础，育特色，现代农业不断壮大。粮食总产6.2亿公斤，继续保持稳定。果品总产20.8亿公斤；合同种植烟叶26.7万亩，收购70万担，均居全省第一。食用菌出口突破1亿元，同比增长10倍，成为全市第一大出口农产品。缘份果业等6个产业化集群进入省名单，规模以上农业龙头企业达到182家，现代农业示范园区88家，登记注册家庭农场60家。成功举办2013中国苹果年会和三门峡苹果财富大会，三门峡果品知名度显著提升。在全国第三家建成农业综合标准化示范市，雏鹰黑猪通过国家生态原产地认证。治理水土流失面积80平方公里，建成62个乡镇防汛视频会商系统，在全省首家实现全覆盖。

　　强引领，重统筹，新型城镇化步伐加快。新一轮城市总体规划纲要通过专家评审，中心城区新增供热面积30万平方米，新建、改建雨水和污水管网30公里，三门峡新污水处理厂建成投用。黄河公园一期建成开放，成为群众休闲娱乐的好去处。三门峡商务中心区完成投资84亿元，8条道路建成，昔日的荒山荒沟，正在成为城市发展新平台。6个特色商业区发展规划全部获批，完成投资43亿元。全市城镇化率48.6%，比上年提高1个百分点。新建、改建农村公路200公里。开工新农村社区住宅1.1万套，完成10个村的新农村建设任务。3个村入选农业部“美丽乡村”试点，4个村入选河南省试点。

　　扩开放，谋合作，发展活力日益增强。深入推进大招商，签约项目153个，合同金额1003亿元。进出口总额2.9亿美元，增长26.7%；实际利用外资8.7亿美元，增长16.3%；实际到位省外资金260.7亿元，增长22.9%。豫晋陕黄河金三角区域合作规划即将获批，首次实现金三角区域合作高层对话，区域凝聚力、影响力日益增强。与中金集团等央企达成合作项目14个，总投资468亿元，全年完成投资64亿元。5家国有银行战略合作协议到位资金231.5亿元，市级投融资平台融资30亿元。与工农中建等16家金融机构开展新一轮战略合作，签约总额1380亿元。中原证券黄河金三角示范区分公司挂牌，广发银行三门峡分行开业。

　　惠民生，促和谐，群众生活持续改善。持续加大民生投入，全市财政民生支出108亿元，占公共财政预算支出的70.2%。省、市十项民生工程全面完成。努力扩大就业，深入实施“崤函创业扶持行动”，发放小额担保贷款7.3亿元，带动2.6万人实现就业和再就业，城镇登记失业率2.86%。提高社会保障水平，城乡低保标准分别提高13%和19%，发放城乡低保金2.1亿元。建成保障性住房8742套，廉租房申请家庭人均月收入标准从550元提高到1020元。加大扶贫开发力度，完成扶贫搬迁6463人，实施整村推进23个，帮助2.2万农村贫困人口稳定脱贫。新解决17万农村人口的饮水安全问题。加快发展社会事业，三门峡社会管理职业学院通过省考核评审，市实验小学迁建、实验中学改扩建项目投入使用。人均基本公共卫生服务经费由25元提高到30元，新农合财政补助资金每人增加50元，市中心医院通过省“三甲”验收。免费孕检实现全覆盖，人口自然增长率4.5‰。崤函古道申遗通过联合国教科文组织验收，“中国公众考古·仰韶论坛”影响深远，三门峡灿烂的文化正焕发出迷人魅力。三门峡党史方志馆建成开馆，市第七届运动会成功举办。“碧水蓝天美丽家园工程”扎实推进，依法取缔中心城区燃煤小锅炉，锅炉整治和机动车污染防治工作走在全省前列。PM2.5监测系统建成并联网发布信息。创建全国绿化模范城市工作扎实推进，卢氏县、灵宝市被评为全国绿化模范县（市），义马市成为全省首个国家水土保持生态文明市，甘山国家森林公园建成国家级生态文明教育基地。强化社会治安综合治理，在全省率先夺得全国社会管理综合治理“长安杯”。安全生产和食品安全形势平稳。妇女儿童、残疾人、老龄、慈善等事业全面发展。

　　转作风，提效能，政府自身建设不断加强。深化行政审批制度改革，取消、下放审批权限15项。严格执行市人大及其常委会的决议、决定，自觉接受市人大及其常委会的法律监督和市政协的民主监督，办理人大代表建议71件、政协委员提案335件。扎实推进“两转两提”，行政效能和公务员队伍素质不断提高。在全省首家试点开展行政执法专项治理，规范执法流程，列出“权力清单”。全面贯彻落实中央“八项规定”和省市意见，全市会务、公务接待、公车使用、公款出国（境）支出分别下降38.3%、34.5%、13.5%和12.4%。

　　一年来，全市上下团结一心、攻坚克难、干事创业、务实发展的氛围日益浓厚，“明理诚信，善做善成”的三门峡精神深入人心，涌现出刘秋玲、马士友等一批道德模范，他们都是“三门峡精神”的楷模。国防教育和国防后备力量建设取得新成效，实现了全省双拥模范城“七连冠”。驻峡解放军、武警部队和广大预备役官兵在支持地方经济建设中发挥了重要作用。统计、审计、外侨、民族、宗教、对台、地震、人防、无线电、黄河河务等工作取得了新成绩。对口援疆工作富有成效。

　　各位代表，转型充满艰辛，成绩来之不易。这得益于省委、省政府和市委的正确领导，离不开市人大、市政协的支持和帮助，凝聚着全市人民的心血和汗水，体现着方方面面的关心和付出。在此，我代表市人民政府，向全市各行各业的建设者，向各位人大代表、政协委员，向驻峡人民解放军、武警官兵、公安民警，向广大客商以及关心支持三门峡发展的各界人士，致以崇高的敬意和衷心的感谢！

　　我们也清醒地认识到，全市经济已进入增速换挡期、结构调整期、改革攻坚期，经济社会发展面临许多困难和挑战：产业结构调整和发展方式转变难度较大；统筹城乡发展任务艰巨；改善民生和环境治理任务繁重，政府自身建设还存在薄弱环节等。对此，我们一定以高度负责的态度，沉着面对，认真解决。

　　二、2014年总体要求和主要预期目标

　　今年是全面深化改革的开局之年，也是落实“十二五”规划的攻坚之年。虽然发展仍面临不少困难和挑战，但世界经济复苏态势趋好，我国经济稳中向好的基本面没有改变，全面深化改革将释放新的动力和活力，国家加快城镇化进程将为中小城市发展带来更多机遇，即将批复的《豫晋陕黄河金三角区域合作规划》将为我市创造更多政策优势，全市转型发展的内生动力正在形成，加上科技革命方兴未艾，新兴业态不断涌现，为全市转型发展提供了重大机遇。我们必须保持清醒，坚定信心，抢抓机遇，以转变发展方式的主动、调整经济结构的主动、改革开放的主动，赢得发展上的主动、竞争中的主动，奋力谱写转型发展、富民强市的崭新篇章。

　　今年政府工作的总体要求是：全面贯彻党的十八大、十八届三中全会精神和市委的决策部署，紧紧围绕“三大战略定位”和“十六字”奋斗目标，继续坚持“求转、求进、求实”总要求，持续深化“四大一高”战略，切实把提高发展的质量和效益放在中心位置，把改革创新贯穿经济社会发展各个领域，在转型升级中加快发展，在深化改革中创新发展，在开放合作中共赢发展，在生态建设中永续发展，在为民惠民中和谐发展，奋力开创经济社会持续健康较快发展的新局面。

　　2014年经济社会发展的主要预期目标是：在提质增效基础上，生产总值增长10%以上；地方公共财政预算收入增长15%以上；规模以上工业增加值增长13%以上；全社会固定资产投资增长21%以上；社会消费品零售总额增长14.5%以上；进出口总额增长9.5%以上；实际利用外商直接投资增长10%以上；城镇居民人均可支配收入和农民人均纯收入均增长10%以上；城镇登记失业率控制在4.5%以内；人口自然增长率控制在6.5‰以内；节能减排完成省定任务。

　　各位代表，转型发展的万里长征，我们刚刚迈出第一步。我们将突出“底线思维”，保持竞争态势，努力追求有质量、有效益、有充分就业、不牺牲环境的速度，让民生改善与发展质量同步提升，让经济总量与白天鹅数量一起增长！

　　三、2014年重点工作

　　（一）以“四大一高”为引领，加快经济转型发展。坚持“四大一高”战略不动摇，走好转型发展之路。

　　持续健全大通关，突出开放新优势。探索“属地申报、属地放行”通关模式，推动海关、检验检疫合作，共享使用查验场。用好“郑新欧”、“郑满欧”铁路货运班列运行机遇，加强与郑州海关、郑州铁路集装箱口岸战略联合，建立陆港物流公司，搭上国际物流班车，努力打造“丝绸之路经济带”的重要节点。

　　持续完善大交通，强化基础新支撑。加快连霍高速改扩建、三淅高速卢氏至西坪段等续建项目进度，积极推进310国道改线，确保蒙西铁路年初开工。加快渑垣高速、运宝高速、三门峡黄河公铁两用桥等项目前期工作。完成省道250线、314线部分路段建设及大中修养护。新建、改建农村公路300公里。

　　持续繁荣大商贸，挖掘内需新潜力。推进环球金融中心、嘉亿城市广场等项目建设，在中心城区重点培育家居建材、小商品批发、农副产品、旅游服务等八大品牌专业市场，形成商务中心区、湖滨特色商业区和中心城区东部三大专业市场群。落实企业工商登记“先照后证”等改革措施，加快限额以上商贸企业和个体工商户发展，年底全市限额以上商贸企业达到320家以上，新增个体工商户等新型创业实体5000家以上。大力实施商标战略，打造一批具有较强市场竞争力和影响力的商标品牌。

　　持续提升大旅游，打造城市新品牌。树立“城市即景区”的理念，以沿黄半岛景观带为主体框架，整合三门峡大坝、虢国博物馆、天鹅湖湿地公园、黄河公园、陕县温泉、天井窑院、函谷关、甘山等旅游资源，强力推进主城区旅游，打造生态休闲旅游目的地。加快推进天鹅湖—函谷关联合创建国家5A级景区步伐，抓好黄河公园二期和天鹅湖湿地公园三期项目，积极建设特色旅游商业街区和大型旅游综合体。完善基础设施和公共服务体系，解决好旅游基础设施“最后一公里”问题。促进旅游业与文化、信息等产业深入融合，办好第20届黄河文化旅游节暨投洽会，深化“沿黄九省（区）黄河之旅旅游联盟”合作，借力大型旅游集团，加大北京等重点市场推介力度，将“黄河三门峡，美丽天鹅城”的品牌叫响全国。

　　持续壮大高新产业，凝聚转型新能量。加强工程实验室、院士工作站等创新平台建设，新认定国家级高新技术企业5家，新增省市级企业研发平台10家，力争实现国家级研发平台“零”的突破。

　　持续推进信息化，促进四化新融合。制定工业化和信息化融合行动计划，引导工业企业利用互联网平台开拓市场，在重点企业开展信息化贯标试点，助推工业转型发展。加快农业物联网建设，建立覆盖全市的农产品质量追溯体系、农业地理信息体系、农机智能指挥调度体系和农业技术推广服务体系。加快智慧城市、智慧旅游建设，逐步实现公共服务区域无线互联网信号全覆盖，推动电子政务及民生和社会领域的信息化应用，让转型发展在信息化的大潮中劈波斩浪、扬帆远航。

　　（二）以“一高两化”为方向，加快工业结构调整。突出工业主导地位，依托五大千亿元产业集群，全年实施超亿元工业项目90个以上，完成工业投资300亿元以上。大力发展先进制造业。持续推进三门峡汽车零部件产业园、骏通公司1万辆起重举升专用车和1万辆多轮驱动专用车、新华水工机械厂搬迁扩建等项目，改造提升化工机械、精密量仪、矿山设备等传统机械工业，加快发展装备制造、汽车及零部件等先进制造业，培育工业升级的核心力量。推进传统产业高端化，调整存量。抓好中金黄金产业园、同人铝业、开祥化工甲醇结构调整等项目，推进产品结构优化。落实工业十快企业和十大技改项目评选奖励政策，鼓励企业加大技改力度，生产质量和性价比更高的产品。淘汰落后产能，确保提前一年完成“十二五”任务。推进高新产业规模化，扩大增量。抓好速达电动汽车、义腾公司涂覆隔膜等一批高新产业重大项目。加快平晟电子科技1200万套液晶显示模块等项目建设，加大电子信息产业招商，引进整机生产项目，促进电子信息产业发展。尽早开工建设汇源集团杜仲饮料项目，加快食品、生物医药产业发展。抓好恒生科技丙尔金、朝晖铜业1万吨压延铜箔等项目，加快新材料产业发展。加快产业集聚区建设。完善基础设施，年内各产业集聚区主干路网全部打通，水、电、暖、污水处理等设施保障到位。实施产业集群培育工程，每个产业集聚区新引进行业龙头企业或投资超5亿元的工业项目2个以上，推进主导产业集群发展。严格土地、环境等准入门槛，促进提质增效。持续强化企业服务。支持优势企业扩大生产，帮助困难企业解困突围，抓好已投产工业项目的服务。加快政府性投资担保机构建设，设立小微企业风险补偿基金，着力解决中小微企业资金难题。继续抓好工业经济运行监测分析，推进大用户直供电试点，建立煤电中长期供需协议，强化要素保障。

　　（三）以扩量提质为途径，大力发展现代服务业。通过改革推动、项目带动和政策引领，促进服务业发展提速、比重提高、水平提升。加快发展高成长性服务业。持续壮大会展经济，办好第三届特博会，引进承办各类专业展会活动，以特博会为龙头，打造一批会展品牌。强力推进现代物流，建设三门峡综合物流园区，成立黄河金三角物流联盟，抓好义煤集团豫西煤炭储配基地等项目建设。着力提升金融业，积极引进股份制银行在我市设立分支机构，支持村镇银行等农村金融机构发展。积极培育新兴服务业。加快发展养老、健康服务业，推进市老年服务中心升级改造，开工市福利院老年养护楼，完善灵宝、卢氏综合性养老服务中心功能，建成陕县、渑池养老服务中心；积极发展康复、护理等医疗机构，满足不同人群的健康服务需求。大力发展电子商务，引进有影响的龙头电子商务企业在我市设立分支机构，支持三门峡农产品电子商务中心等平台建设，探索发展电子商务产业园。鼓励支持商贸实体企业建立网店，培育一批家庭网店。加强网络市场监管，规范网络交易行为。改造提升传统服务业。引入现代管理理念、新型商业模式和信息技术，推动运营模式、销售渠道、服务方式等创新，推动商贸流通等传统服务业转型升级。加快推动二、三产业分离试点工作。持续繁荣房地产市场。

　　（四）以增收富民为重点，加快发展现代特色农业。加大强农惠农力度，稳定粮食生产，壮大特色农业，促进农民增收。提升特色农业质量和效益。抓好粮食生产，总产保持在5亿公斤以上。继续发展特色果品业，新栽植果树30万亩，确保总产20亿公斤。大力发展生态养殖，推进规模养殖场集约化、标准化改造。持续抓好烟叶生产，发展中药材30万亩、食用菌1.5亿袋。培育壮大现代农业产业化集群。打造2—3个示范性农业产业化集群，争取进入下一批省农业产业化集群名单。实施农业示范园区引领工程。在中心城市和县城周边，积极发展都市农业，规划建设一批兼具城市“菜篮子”、生态绿化、休闲观光等综合功能的现代特色农业园区。构建新型农业经营体系。大力培育龙头企业、专业大户、家庭农场、农民专业合作社等新型农业经营主体，新发展家庭农场90家，农民专业合作社达到850家以上，现代农业示范园区达到120家以上。加强农产品品牌建设。新认证无公害农产品、绿色食品、有机食品和地理标志保护产品10个以上。改善农村生产生活条件。完善补贴制度，提高补贴精准性、指向性。加快实施大石涧水库等重大水利工程，完成12座小Ⅱ型水库除险加固和5条中小河流治理。对20个贫困村实施整村推进扶贫开发，再实现2万农村贫困人口脱贫。新增沼气工程20座，再解决10万农村人口饮水安全问题。进一步做好农业防灾减灾工作。

　　（五）以“人的城镇化”为核心，科学推进新型城镇化。按照以人为本、优化布局、生态文明、传承文化、产城互动、科学有序的原则，积极稳妥推进新型城镇化。优化城镇化体系布局。全面完成城市总体规划修编，完成城乡一体化示范区建设实施方案、城市综合交通体系和城市道路等专项规划。按照“两大三小”组团式布局，构建中心城市、县（市）城区、小城镇和“美丽乡村”层次分明、协调发展、独具特色的新型城镇化体系。年内，全市城镇化率提高1个百分点以上。强化中心城区带动战略。促进湖滨城区、商务中心区和陕县城区、产业集聚区融合发展，抓好城乡一体化示范区建设，以“两大”促“三小”，形成分工合理、功能互补、联动发展的格局。加快中心城区“两轴线一中心”区域改造，把中心城区打造成引领区域发展的“主引擎”。抓好“三小”组团建设。持续实施义渑城区对接规划，逐步实现基础设施、公共服务、产业配套等一体化。加快灵宝市城区改造和建设，向东形成与三门峡城乡一体化示范区对接的态势。继续改造和美化卢氏老城区，加快洛河南岸开发建设，打造“生态宜居特色新山城”。统筹推进小城镇和“美丽乡村”建设。分类推进小城镇建设，强化产业支撑，完善基础设施和配套服务，因地制宜发展重点城镇。加强农村人居环境综合整治，推进“美丽乡村”建设。抓好产业载体建设。坚持产业为基、就业为本，促进产业集聚区“产城融合”，加快城市公共设施向产业集聚区延伸，每个产业集聚区新增就业岗位1000个，让转移农民“进得来，落得下”。加快商务中心区建设，80栋在建楼房年内封顶，基础设施全部建成，实现“三年成规模”目标。加快各县（市、区）特色商业区建设，积极引进城市综合体，培育一批金融服务、文化创意、总部经济等特色楼宇，完成投资60亿元，新开工项目10个，实现营业收入70亿元。提升城市公共服务水平。中心城区新增4条公交线路，加快推进400辆出租车更新及新增步伐。统筹抓好水、电、暖、污水垃圾处理等基础公共服务。合理布局，加快中心城区公厕建设，并实行免费开放。抓好保障性安居工程，开工保障性住房5797套，将符合条件的农业转移人口纳入城镇住房保障体系。开展“城市管理年”活动。抓好城区净化、绿化、亮化、美化，提高城市精细化管理水平，让城市更加宜居、社会更加有序、生活更加便利。创新城镇化工作体制机制。深化户籍制度改革，有序推进农业转移人口市民化，推动城中村、城郊村和产业集聚区的农业人口成建制转为城镇户口。创新土地管理，提高城镇建设用地效率。创新融资机制，用好市、县投融资平台和政策性金融机构的融资服务。各位代表，推进新型城镇化，是一项长期而艰巨的任务，我们要积极有为，扎实工作，努力建设“望得见山，看得见水，记得住乡愁”的美好家园！

　　（六）以重点项目为抓手，增强转型发展后劲。实施重点项目280个左右，总投资2000亿元以上，年内完成投资550亿元以上。推进项目开工。确保灵宝华鑫1万吨电解铜箔技术改造等144个项目按计划开工建设。加快建设进度。抓好中金集团黄金冶炼厂搬迁改造等139个在建项目服务保障，确保项目尽快建成。抓好项目储备。抓住中央下放审批权限的机遇，围绕国家支持的重点领域，谋划储备80个亿元以上项目，增强转型发展后劲。激活民间投资。落实促进民间投资、发展非公有制经济的政策措施，筛选一批重大项目，吸引民间资本参与。鼓励社会力量提供医疗卫生、教育、文化、社区服务等公共服务，充分发挥市场在资源配置中的决定性作用，让各类市场主体活力竞相迸发，让一切创造财富的源泉充分涌流。

　　（七）以招商合作为载体，提升开放型经济发展水平。加大招商引资力度，确保全年签约金额1000亿元以上。拓宽开放领域。放宽外资市场准入，努力在大型农业龙头企业、商贸企业、社会事业招商上实现新突破，构建全方位、多层次、宽领域的开放格局。创新招商方式。积极开展驻地招商、以商招商、中介招商，有针对性地参加各类招商活动，注重发挥企业在招商中的主体作用和商会协会的桥梁作用，有重点地寻找潜在客商，提高招商引资的实效性。提升招商质量。重落地、重投产、重带动，大力引进竞争力、成长性和关联性强的项目，积极引进先进技术、人才、机制、管理。加强已签约项目的跟踪督导，争取早日开工。优化开放环境。继续优化政务、服务和施工环境，为项目和企业提供“保姆式”服务。拓展外需市场。充分利用大通关平台，加大出口基地建设，巩固果汁等产品出口，培育食用菌、专用汽车等新的出口增长点。强化区域合作。加快推进黄河金三角地区基础设施对接，合力承接发展装备制造业，共同培育战略性新兴产业，努力把黄河金三角地区打造成全国区域经济的新生增长极。

　　（八）以深化改革为突破，释放发展的动力和活力。切实抓好各项改革任务的落实，努力在重要领域、关键环节取得实质进展。扎实推进财税体制改革。深化政府和部门预决算公开，建立跨年度预算平衡机制。清理整合专项转移支付项目，统筹整合专项资金，提高财政资金使用效益。清理规范税收优惠政策，全面落实营改增、资源税从价计征等改革政策。加强预算绩效管理，盘活财政存量资金。加强政府债务管理，有效防范财政风险。深化金融改革。健全金融机构体系，加快金融产品和服务模式创新。完善各级投融资平台功能，提升投融资能力。优化金融生态环境，完善投融资担保体系，严厉打击非法集资，防范和化解金融风险。推进企业上市，争取更多企业进入省上市“后备队”。深化农村改革。落实农村土地集体所有权，稳定农户承包权，放活土地经营权，探索增加农民财产收益的有效途径，增强农民向城镇转移的动力。加快农村集体建设用地和宅基地确权登记，探索宅基地有偿退出机制。加快集体经济股份制改革，建设农村集体产权、林权和“四荒”交易平台，推动产权要素合理流动。统筹推进经济领域和其他领域改革，努力完成中央、省改革任务。

　　（九）以保障民生为目的，努力让群众过上更好生活。把促进社会公平、增进民生福祉作为政府工作的出发点和落脚点，全力办好民生实事，让发展成果更多、更公平惠及全市人民。努力实现高质量就业。把就业作为最大的民生，积极推进就业服务平台建设，继续实施“崤函创业扶持行动”、农民工职业技能提升计划，重点抓好高校毕业生、农村转移劳动力、化解产能过剩中出现的下岗失业人员就业，新增城镇就业4万人以上。深入实施“崤函人才计划”，为发展提供人才支撑。完善城乡社会保障体系。继续扩大社会保障覆盖范围，加强城镇企业职工养老保险与城乡居民养老保险衔接，探索城镇职工医保与居民医保、新农合的衔接，加快实施社会保障“一卡通”。加强社会保险与国有商业保险合作，抓好乡镇社会保障平台建设，把社会保障的“网底”编实、筑牢。大力发展社会事业。落实好义务教育均衡发展推进计划，适应新型城镇化发展，统筹抓好学前教育，解决好幼儿园“大班额”问题。加快推进职教攻坚二期工程，抓好三门峡社会管理职业学院建设。统筹安排好进城农民子女就近入学、平等接受教育。做大做强文化产业，加大文化遗产保护力度，开工建设庙底沟遗址文化公园。推进政府采购百场戏、数字化图书馆等文化惠民工程建设。继续深化医药卫生体制改革，建设市中心医院全科医生临床培养基地和影像中心，探索建立区域医疗联合体，优化医疗资源配置，更好地保障百姓健康。调整完善生育政策，促进出生人口性别比平衡和素质提高。加强环境保护和生态建设。落实国家大气污染防治措施，铁腕整治工业废气、施工及道路扬尘、机动车尾气、秸秆焚烧等污染，加大雾霾天气治理力度。开展南涧河、弘农涧河水污染专项治理，加强南水北调水源地等重点饮用水源地保护，确保全市饮水安全和一泓清水送北京。抓好重金属污染防治。扎实推进全国绿化模范市创建工作，新增造林面积28.8万亩，做好黄河湿地生态系统的修复和恢复，加强白天鹅保护，通过持续不懈的努力，守护绿水青山，留住蓝天白云。加强社会治理创新。加强社会管理综合治理，扎实做好信访工作，排查化解社会矛盾，积极回应基层要求和群众呼声。做好第八届村委会换届选举工作。深入推进公民道德建设，广泛开展“做文明人、办文明事”主题实践活动。持续开展“扫黄打非”，加强网络文化建设和管理。以更加坚决、更加有力、更加有效的措施，加强安全生产监管，坚决遏制重特大事故发生。健全食品药品安全监管体系，用严格的标准、严密的监管、严厉的惩处、严肃的问责，确保群众“舌尖上的安全”。强化应急管理体系建设，提高应急处置能力。强化平安建设，依法打击违法犯罪活动，维护社会大局和谐稳定。

　　继续加强国防动员、国防教育和国防后备力量建设，支持驻峡解放军、武警部队、民兵、预备役和人民防空建设，深入开展双拥共建，融洽军民鱼水情。支持工会、共青团、妇联、工商联等人民团体发挥桥梁纽带作用。继续做好外侨、对台、民族、宗教、史志、气象、地震等工作。搞好第三次全国经济普查。继续做好对口援疆工作。

　　四、加强政府自身建设

　　深入推进“两转两提”，实施“政府服务质量提升年”行动，加快建设法治政府和服务型政府。

　　加快转变政府职能。深入推进简政放权，继续清理行政审批事项。落实统一的市场准入制度，实行负面清单管理模式。强化政府公共服务、市场监管、社会管理、环境保护等职责，推动政府职能加快向创造良好发展环境、提供优质公共服务、维护社会公平正义转变。适应职能转变要求，优化政府机构设置、职能配置，全面完成政府机构改革任务。

　　深入推进依法行政。认真执行市人大及其常委会决议决定，自觉接受法律和工作监督，主动接受市政协和各民主党派的民主监督，认真办理人大代表议案、建议和政协委员提案。坚持依法行政，理顺执法体制，推行服务型行政执法，规范公正文明执法。深入推进普法宣传，增强全社会法治意识。严格行政问责，深化政务公开，让人民监督权力，让权力在阳光下运行。

　　切实改进工作作风。认真落实中央八项规定，深入开展群众路线教育实践活动，着力解决“四风”问题。加强廉政建设，完善惩防体系，强化纠风和专项治理。坚持从严从俭，健全厉行节约反对浪费制度体系，严格控制一般性支出，压缩“三公”经费，在政府过紧日子中让人民过上好日子！

　　各位代表，梦想承载希望，奋斗开创未来。让我们在省委、省政府和市委的坚强领导下，在市人大、市政协的监督支持下，敢于担当，奋发进取，为加快建设创新开放、富裕文明、平安和谐、生态宜居三门峡而努力奋斗！
